# A Domain Adaption ResNet Model to Detect Faults in Roller Bearings Using Vibro-Acoustic Data

**DOI:** 10.3390/s23063068

**Published:** 2023-03-13

**Authors:** Yi Liu, Hang Xiang, Zhansi Jiang, Jiawei Xiang

**Affiliations:** 1 School of Mechanical and Electrical Engineering, Guilin University of Electronic Technology, Guilin 541004, China; 2 School of Mathematics and Computer Science, Northwest Minzu University, Lanzhou 730000, China; 3 College of Mechanical and Electrical Engineering, Wenzhou University, Wenzhou 325035, China

**Keywords:** intelligent fault diagnosis, roller bearings, multi-source data, domain adaption, ResNet

## Abstract

Intelligent fault diagnosis of roller bearings is facing two important problems, one is that train and test datasets have the same distribution, and the other is the installation positions of accelerometer sensors are limited in industrial environments, and the collected signals are often polluted by background noise. In the recent years, the discrepancy between train and test datasets is decreased by introducing the idea of transfer learning to solve the first issue. In addition, the non-contact sensors will replace the contact sensors. In this paper, a domain adaption residual neural network (DA-ResNet) model using maximum mean discrepancy (MMD) and a residual connection is constructed for cross-domain diagnosis of roller bearings based on acoustic and vibration data. MMD is used to minimize the distribution discrepancy between the source and target domains, thereby improving the transferability of the learned features. Acoustic and vibration signals from three directions are simultaneously sampled to provide more complete bearing information. Two experimental cases are conducted to test the ideas presented. The first is to verify the necessity of multi-source data, and the second is to demonstrate that transfer operation can improve recognition accuracy in fault diagnosis.

## 1. Introduction

Bearings as the transmission parts are installed in rotating machinery that will affect the safety of the whole rotating equipment system [1,2]. The little defect of the bearing will cause heavy disaster and even more casualties during the operation. Therefore, the safety monitoring and fault diagnosis of the rotating equipment system has become a hot topic in recent years.

The transformer winding defects always affect the safety of the power grid and power equipment [3,4,5]. Bearing is a vital part to realize power transmission and load in rotating machinery. Bearings have a high failure ratio compared with other moving parts during machinery operation. To eliminate the life-threatening dangers in industrial production in recent years, the existing methods of bearings have been developed by many researchers and experts. For example, the time domain, frequency domain and time—frequency domain of traditional fault diagnosis methods are effective to extract fault-related features of vibration signals [6,7,8,9]. However, the accuracy of fault diagnosis will be affected by the rotating machinery complexity. On the other hand, the collected massive signals cannot be analyzed one by one as it is an extremely time-consuming process. Intelligent fault diagnosis methods of bearings have attracted many professional researchers’ eyes, and the development of these ways is becoming more integrated. The nonlinear relationship between vibration signals and fault categories is characterized by the machine learning technique, the conventional fault methods’ clustering analysis for fault diagnosis of winding fault type [10]. For example, principal component analysis (PCA) [11], support vector machine (SVM) [12], and artificial neural network (ANN) [13,14]. However, complex measurement environments and operating conditions will lead to the acquisition of polluted data making the mentioned methods almost ineffective. Deep learning is an enhanced machine learning method to solve the above problems. The internal correlations and hidden details of the signal are exposed by deep learning methods with deep and complex network structures [15,16]. In addition, the complex mapping relationship also can be characterized. The common methods include the auto-encoder (AE) [17], long short-term memory (LSTM) [18], generative adversarial networks (GAN) [19,20], and the convolutional neural network (CNN) [21,22,23].

Nevertheless, these data-driven fault diagnosis methods are applied to single-sensor vibration signals in constructed models [24,25,26]. The restriction of coverage range and installation location meant that the accelerometer could not measure all the information of the machinery equipment to monitor the health status. Acoustic signal analysis is usually used to localize faults and diagnose faulty bearings. The acoustic is conducted by the object vibration. The acoustic is another expression form of vibration. Omoregbee handled acoustic signals as the input of the improved support vector machine (SVM) model for fault identification [27]. In [28], vibration and acoustic signals are simultaneously sampled to detect the liner scuffing fault in the engine system. Microphones are used to acquire the acoustic signals, and then they are input into the 1D CNN-based networks for fault diagnosis [29]. However, the microphone is yielded to the Doppler effect when the railway vehicle moves very fast; thus, the sampled acoustic signal is polluted by the unrelated components. For this phenomenon, researchers proposed an effective method to remove the Doppler effect embedded in the acoustic signal [30]. 

Vibro-acoustic fault diagnosis methods of bearings have been investigated by many researchers. Ye had detected vibro-acoustic characteristics in axial piston pumps under varying operation conditions [31]. The new damage index is constructed to estimate the nonlinearity of modulated signals to detect the crack width by processing vibro-acoustic signals. The performance of the vibro-acoustic modulation method is better than the PZT-enabled active sensing method in eliminating the saturation phenomenon [32]. In [33], vibro-acoustic signals are used to complete information on fault characteristics for fault diagnosis using an improved fusion algorithm. Subsequently, the features are extracted by a one-dimension convolutional neural network (1D-CNN) from the vibration and acoustic signals, and then achieve 100% recognition accuracy under four speeds. Yang et al. combined two improved projection methods for fault diagnosis, in particular the sampling of vibro-acoustic signals under various operating conditions [34]. To eliminate the frequency smearing phenomenon and expose the fault characteristic frequency in the envelope spectrum, a new transient signal analysis (TSA)-based angular resampling method was proposed for fault diagnosis under variable speed conditions by sound signal analysis [35].

However, no matter whether the data are sampled from a single-sensor or multi-sensors, they still need to solve the insufficient labeled data problem. Massive data are labeled manually, and this operation needs a huge manual operation and relies on knowledge dependence. Furthermore, the fault diagnosis accuracy is directly affected by the sufficient labeled data. Sometimes, the bearing fault types could be acquired by simulation in a laboratory; this technique could alleviate the shortage of labeled data. Another important factor of the diagnosis accuracy effect is that the distribution of training and testing data is the same. The success of the intelligent fault diagnosis of rotating machinery was demonstrated in [36], which validates the importance of the probability distribution between training and testing sets.

Recently, a powerful tool named transfer learning has been used to solve the distribution discrepancy of intelligent fault diagnosis areas [37]. The difference between classical intelligent fault diagnosis and transfer learning is that the latter has two datasets, source domain and target domain. Without a doubt, source domain distribution is different from the target domain. Reducing the distribution discrepancy is the purpose of transfer learning, which will apply the knowledge of the labeled data to enhance the predictive model performance to identify the unlabeled data accurately. The feature-based method is proposed to achieve the goal of distribution discrepancy reduction. The transferable features can be learned by the deep hierarchical model from the cross-domain data. The model is automatically learning features, which reduces the time cost compared with feature mapping. In computer vision and speech recognition areas, feature-based methods are widely employed and yielded some achievements [38,39]. The method provided a new idea that the source domain data consists of spectrum data and partially labeled target domain data [40]. MMD is an index to check whether two datasets are from the same distribution. Domain adaption enhanced the deep convolution neural network to implement fault diagnosis under different noise levels [41]. 

It is worth considering that accelerometer sensors, installed on flat positions of the equipment surfaces, could obtain machine-related information for fault diagnosis. The contact sensors are not suitable in irregular positions. Non-contact sensors are very suitable to sample the above working environments. An acoustic signal is sampled by the microphone, which is a non-contact sensor. On the other hand, source and target data distribution discrepancy is minimized by the MMD, and then high diagnosis accuracy will be obtained by transferring the source data knowledge to the target data. MMD is used to minimize the distribution discrepancy of the source and target domains to improve the transferability of the bearing-related knowledge for acquiring high diagnosis accuracy. Meanwhile, transfer learning operations can mitigate the insufficient datasets of bearings. The common problem should be mentioned. As the network depth increases, the difficulty of training the CNN model will gradually increase as well. Meanwhile, adding more layers will bring more large training errors. The ResNet model could solve the problem of the accuracy decrease as a result of the network depth increase by designing identity mappings based on ordinary CNN. Facilitating the backpropagation of errors and optimizing model parameters at the same time. The novelty and contributions of the paper can be concluded as follows:

(1) Acoustic and three directions vibration signals are simultaneously sampled to be regarded as the input of the model to reinforce the diagnosis knowledge of bearings.
(2) MMD is introduced to minimize the distribution difference between source and target domains, thus improving the transferability of learned features. Combining the advantages of the ResNet framework, it can guarantee high recognition accuracy from one defect degree to another defect category.

The remainder of this paper is as follows. Both ResNet and MMD backgrounds are shown in Section 2. Then the procedure of the proposed model is given in Section 3. Section 4 analyzes the necessity of multi-source data and experimental results of the transfer task. Lastly, Section 5 displays the overwhelming conclusion of the article.

## 2. Materials and Methods

### 2.1. MMD Definition

Source and target domains’ dataset distribution discrepancy is measured by MMD. In the transfer learning area, MMD frequently qualifies the similarity of source and target domains. For example, the datasets $U={\left\{u_{i}\right\}}_{i=1}^{n_{s}}$, $V={\left\{v_{i}\right\}}_{i=1}^{n_{t}}$ follow the probability distribution *p* and *q*, respectively. Thus, the definition of MMD can be given:

(1) $$D_{H}(U,V):=\underset{\varphi \in H}{\sup}(E_{U\sim p}\left[\phi (x)\right])-E_{V\sim p}\left[\phi (y)\right])$$

where *H* is reproducing the kernel Hilbert space, and ∅(•) represents the nonlinear mapping, which is from the original feature space to the reproducing kernel Hilbert space. To acquire the maximum distance between datasets *U* and *V*, the low dimension datasets will be mapped in a high dimension space. Based on the kernel mean embedding of distribution, Gaussian kernels acquire reproducing the kernel Hilbert space. The specific formula of MMD is shown as follows:

(2) $$D_{H}^{2}(U,V)={\left\|\frac{1}{n_{s}}\sum_{i=1}^{n_{s}}\phi (u_{i})\frac{1}{n_{t}}\sum_{i=1}^{n_{t}}\phi (v_{i})\right\|}^{2}$$

### 2.2. ResNet

Accuracy will maintain a certain value and degrade rapidly as the network depth increases [42]. This phenomenon is testified by He et al. who had validated that the result is not caused by overfitting. Meanwhile, more layers are added, which will bring larger training errors. 

The structure of the ResNet is composed of the input layer, the convolution layer $f_{conv}$, the residual block, the max-pooling layer $f_{pool}$, the activation layer $f_{relu}$, and the output layer. Aiming at describing the related information of the ResNet; we assume that the sample is $X={\left[x_{1},x_{2,}x_{3},\ldots x_{N}\right]}^{T}$, and the sample’s mean and variance are denoted as

(3) $$\mu (X)=\frac{1}{D}\sum_{j=1}^{D}x_{j}$$

(4) $$\sigma (X)=\sqrt{\frac{1}{D}\sum_{j=1}^{D}{\left(x_{j}^{2}-\mu (D)\right)}^{2}}$$

**Figure 1:**
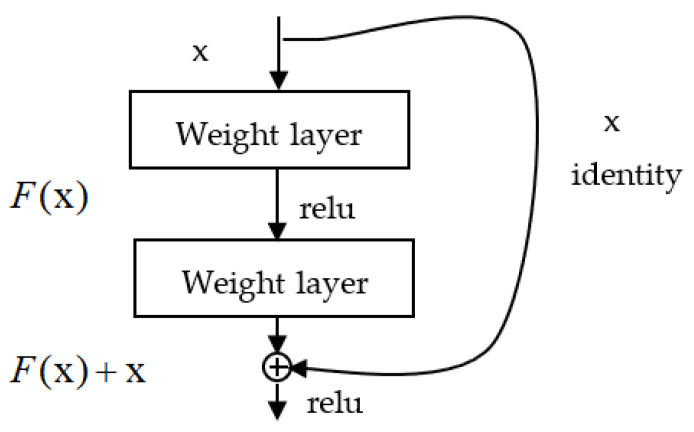
Basic structure of a residual block.

A residual block is introduced to solve the problem of the accuracy decrease as the network depth increases. Furthermore, the residual block can keep the performance of models with the depth of the model increasing. Figure 1 shows the basic structure of a residual block; nowadays, many improved ResNet frameworks always change the basic residual block. The residual block is different from most deep models in that the convolutional layers are connected by skipping, as shown in the curve of Figure 1.

From the above figure, x is the input layer; the output result F(x) is obtained after the linear processing and activation of the first layer. The shortcut connection operation is to add x before the output value of the second layer is activated.

## 3. The Proposed Model of DA-ResNet

**Figure 2:**
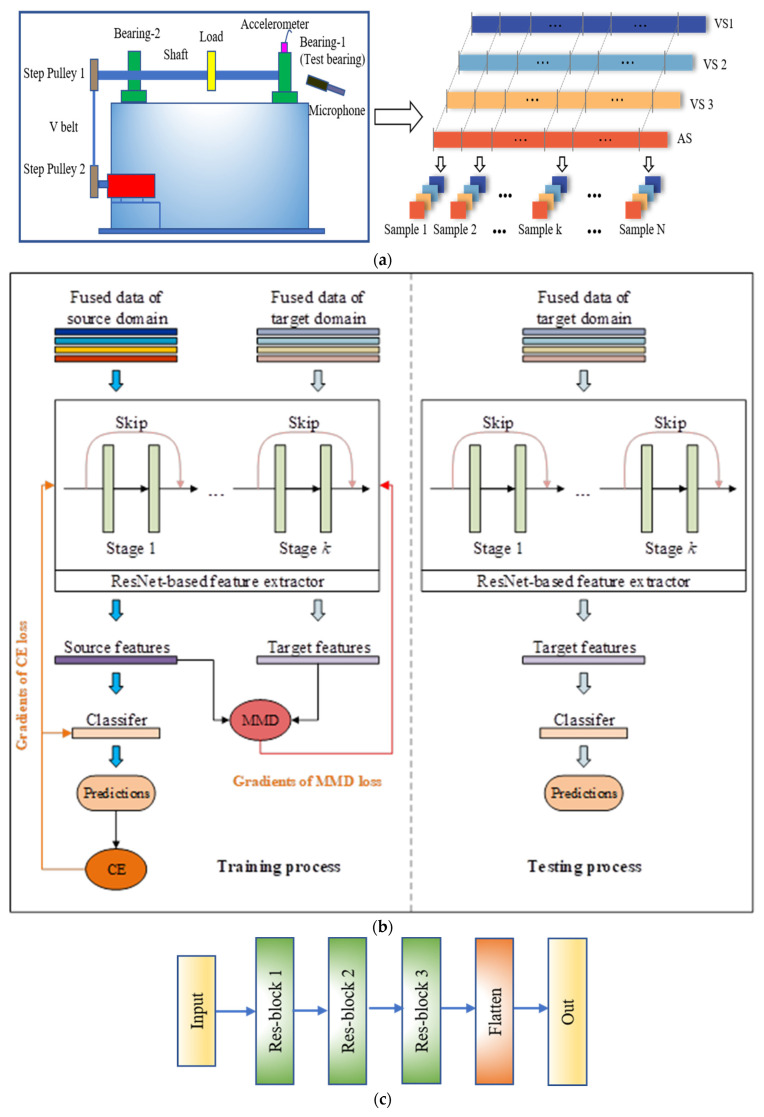

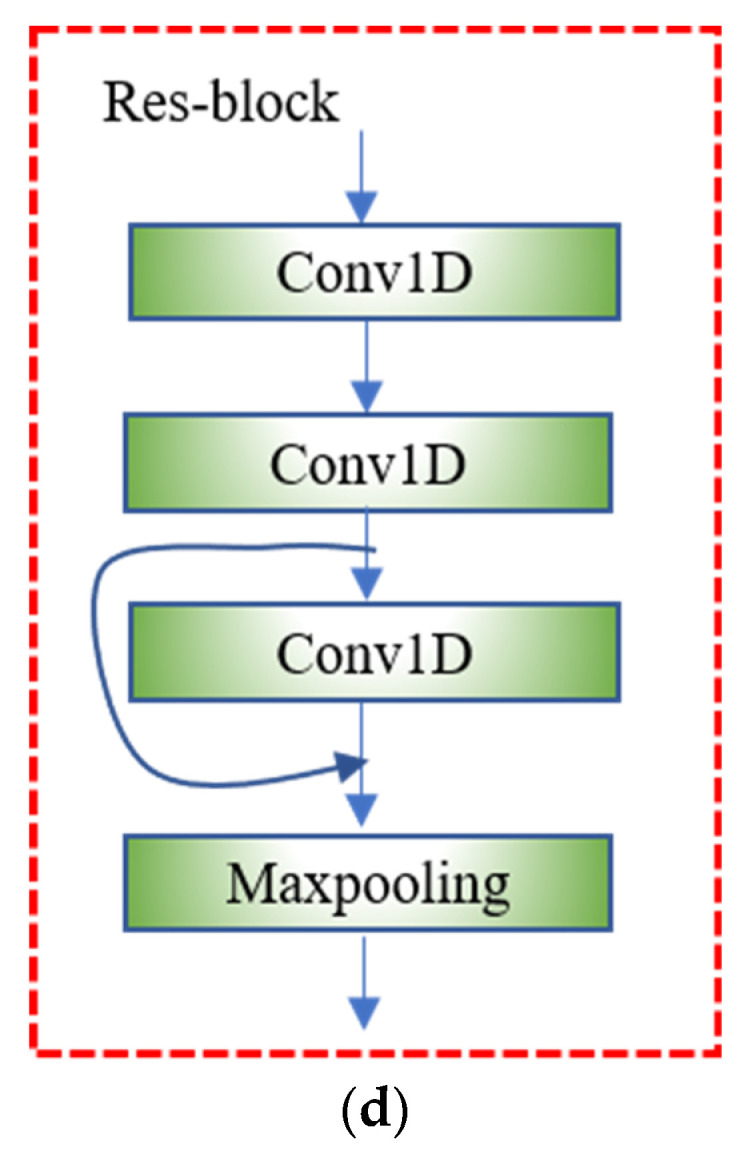
Structure of the proposed method. (**a**) multi-source data; (**b**) cross-domain fault diagnosis; (**c**) structure of ResNet; (**d**) structure of Res-block.

In this paper, two ideas will be validated by vibration and acoustic (vibro-acoustic) data, and their specific processing procedures are shown in Figure 2. In Figure 2a, a typical photograph of the test rig is provided on the left of the data. The accelerometer sensor and microphone are installed nearest to the test bearing to acquire as much rich machinery information as possible. The vibration and acoustic signals are described as VS and AS, respectively. Note that the footmarks of the VSs represent three directions of the accelerometer sensor, vertical, horizontal, and axial directions, which are connected to channel 1, channel 2, and channel 3, respectively. The sample length is set to 4096 and the number of datasets is 960.

In this paper, a ResNet based on domain adaption is proposed for validating the necessity of multi-source data, and the transfer operation is capable of accuracy improvement in fault diagnosis. Commonly, this method consists of four stages: domain partition, data feature extraction, domain adaption, and fault identification. In the first stage, the diagnosis knowledge is obtained by labeled data from the source domain, which is used to identify fault types of the unlabeled data in the target domain

This section may be divided into subheadings. It should provide a concise and precise description of the experimental results and their interpretation, as well as the experimental conclusions that can be drawn.

Second, nonlinear feature mapping is a key processing function, thus, the transferable features can be obtained by data from the source and target domains. It is worth stating the same nonlinear feature mapping is simultaneously applied to the data from the source and target domains. Next is domain adaption. MMD is used to measure the distribution discrepancy of the learned transferable features. Subsequently, the calculated distribution discrepancy of the learned transferable features is considered as an optimization objective to backpropagate, and then to train the parameters of the nonlinear feature mapping. To achieve this purpose, the cross-domain discrepancy of the features needs to be small; in other words, the distribution discrepancy of the learned transferable features is minimized. In the last stage, the unlabeled samples can be recognized by the domain-share classifier. It is noted that the distribution of the learned features of data in the source domain is mastered by the domain-share classifier. After processing by the training with domain adaption, the distribution discrepancy of the learned transferable features from the source and target domains is very small. Finally, the diagram of the cross-domain fault diagnosis is given, as shown in Figure 2b.

The diagnosis procedure of the DA-ResNet model is introduced in the above parts. Then we will introduce the structure of the ResNet framework. The model specification includes an input layer, a convolutional layer, three max-pooling layers, and three residual blocks, the following are flattened and dropout layers. The input of the network is the multi-source signal which includes vibration and acoustics signals. The purpose of the convolutional layer is to get the feature maps, and the weights of the convolutional kernels are allocated over the input. Thus, the number of the required train parameters is significantly deduced. The residual block includes two convolutional operations, ReLU activation functions, and one identity shortcut, as shown in Figure 1. We show the improved ResNet structure in Figure 2c.

## 4. Experimental Verification

### 4.1. Datasets Introduction

**Figure 3:**
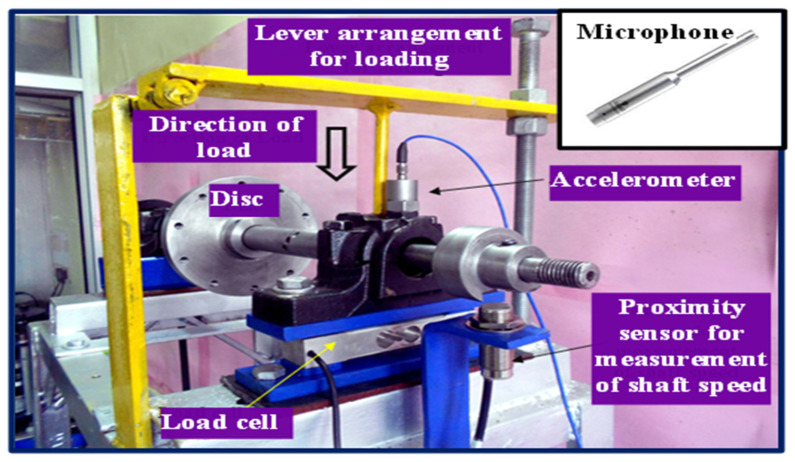
Test rig.

This experiment was conducted in the Precision Metrology Laboratory, at the Mechanical Engineering Department of Sant Longowal Institute of Engineering and Technology Longowal, India. In this case, we try to identify fault types for testifying the property of the DA-ResNet model using the laboratory cylindrical roller bearings with different defect sizes. The test rig is shown in Figure 3, and the shaft speed is measured by the proximity sensor. The power is provided by a 346-Watt AC motor and then is transferred to the shaft; a 2 kg disc is mounted in the middle of the shaft. A device named a lever arrangement is applied to load a roller bearing in the vertical direction. The load cell is installed below the bearing housing to measure the applied load. The accelerometer is set on the top of the bearing housing to decrease the effect of the transferring path. At the same time, the microphone is set to the nearest of the test bearing. The experiment was conducted under the conditions: shaft speed and vertical load are 2050 rpm and 200 N, respectively, and the signals acquired in this work were recorded at a sampling rate of 70,000 Hz.

**Figure 4:**
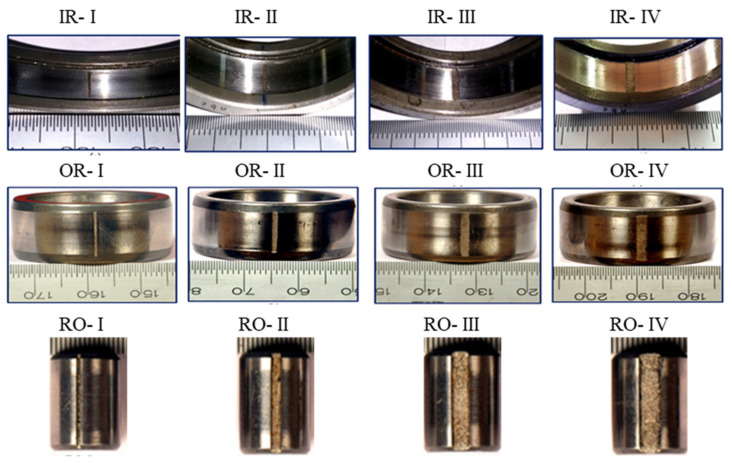
The figures of defect elements of bearings under different fault degrees.

Table 1 and Table 2, respectively, describe the size parameters of the roller bearings and the four failure degrees of inner race, outer race, and roller. The four failure degrees are used to testify the proposed ideas, and the data are divided into four datasets. We labeled the different failure degrees with different numbers for constructing the source and target domains. The figures of the failure elements’ degrees are displayed in Figure 4.

**Figure 5:**
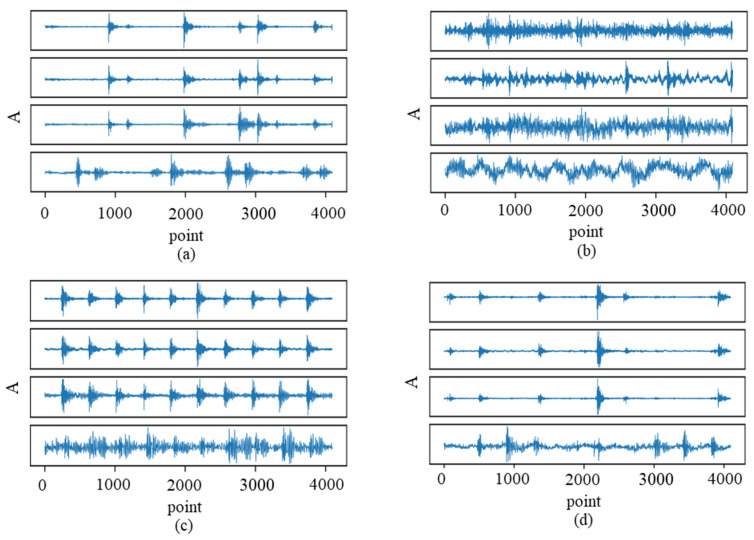
Waveform of vibration and acoustic signals. (**a**) inner race fault; (**b**) normal; (**c**) outer race fault; (**d**) roller fault.

The waveform of vibration and acoustic signals is shown in Figure 5. The *x* axis represents the number of sampled points, and the vertical direction is the amplitude of the vibration and acoustic signals. It is noted that, from top to bottom, are the exhibited subfigures of Figure 5a, named VS1, VS2, VS3 (vertical, horizontal, and axial directions of the tested bearings), and AS signals. The faulty vibration signals have transient impulses, and an inner race and roller cases; the acoustic signals could match the impulses’ locations sometimes. There are two ideas verified by the above signals, the first idea is to check that the DA-ResNet is superior to the other intelligent diagnosis methods. Then, the effectiveness of vibro-acoustic multi-source signal is testified by a transfer task.

### 4.2. Experimental Configuration

The common compared models are used to test the performance of the proposed model. Multilayer perception (MLP), also named ANN, inputs and outputs layers; many hidden layers are included between the input and output layers. The simplest network has a hidden layer, which can learn features from the input data. BiLSTM consists of the forward and backward LSTM, the former is to process the input data and the latter for the reversed data, and then to splice the output of two LSTMs after processing. CNN is a supervised learning neural network with a convolutional layer, a pooling layer, a batch normalization layer, and activation function, commonly regarded as a feature extractor. CNN has had great success in high dimension, such as image, video, and light fields. Low dimension includes seismic waves, radar data, biological signals, and so on. In particular, CNN is widely applied in fault diagnosis for feature extraction.

(1) Baseline: MLP

As a baseline model, the MLP is composed of two dense layers (called fully connected layers). A large number of parameters in MLP results from the full connection between the input and output of each dense layer, and the dropout layer is used to overcome the overfitting caused by numerous trainable parameters in the MLP model. Specifically, the structure of MLP can be described as: {Input (4096,), dense (32,), dropout (32,), dense (128,), dropout (128,)}.

(2) BiLSTM

For the BiLSTM model, the convolutional layer is introduced to overcome the computational complexity caused by the recurrence mechanisms of LSTM and the solving technique is to embed the row signals into a low dimensional feature vector. The structure of BiLSTM is as follows: {Input (4096,), convolution (128, 64), BiLSTM (32,), dropout (32,), dense (128,), dropout (128,)}.

(3) CNN

In CNN, the structure of this model is to stack in turn several convolutional and max-pooling layers. Specifically, the details of the CNN model are as follows: {Input (4096,), convolution (1024, 4), max-pooling (512, 4), convolution (128, 8), max pooling (64, 8), flatten (512,), dense (128,)}.

(4) ResNet

For the ResNet, residual blocks are significant characteristics and provide a multi-receptive field due to the skip-connection. Inspired by residual networks in computer vision, a simple ResNet is designed to diagnose bearings’ faults. The constructed structure of the model can be described as follows: {Input (4096,), convolution (1024, 4), residual block (512, 8), max-pooling (256, 8), residual block (256, 16), max-pooling (128, 16), residual block (128, 32), max-pooling (64, 32), flatten (2048,), dense (128,)}.

In this paper, the specific parameters of the DA-ResNet model are shown in Table 3, withthe improved ResNet in Figure 2c. The specific layers’ parameters of the mentioned four compared models are described in the above part of the table. Similarly, more information on these models is shown in Table 4.

### 4.3. Cross-Domain Fault Diagnosis

To further validate the property of DA-ResNet, the following methods for comparison tests were used,. Methods include MLP, BiLSTM, CNN, ResNet, and domain adaption CNN (DA-CNN). The first four are common deep learning methods for object detection, object recognition, and so on. In this case, the effectiveness of the feature transfer is testified with four experimental tasks. Vibration and acoustic signals are collected under the same working conditions. The difference is the defect size of the faulty elements of roller bearings, and the training sample consists of vibration and acoustic signals. 

**Figure 6:**
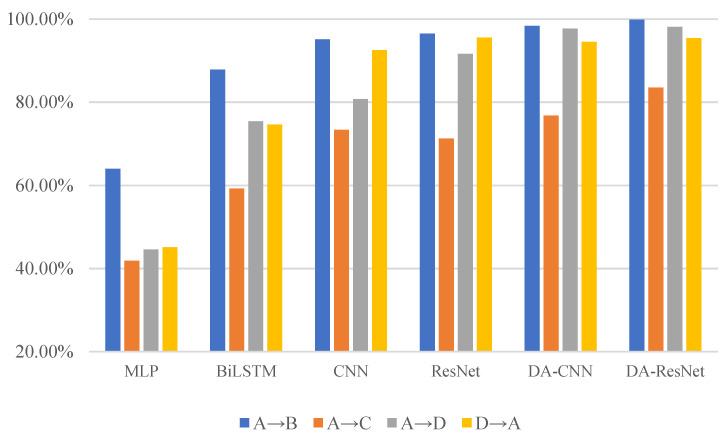
The accuracy of the six methods on various transfer diagnostic tasks.

**Figure 7:**
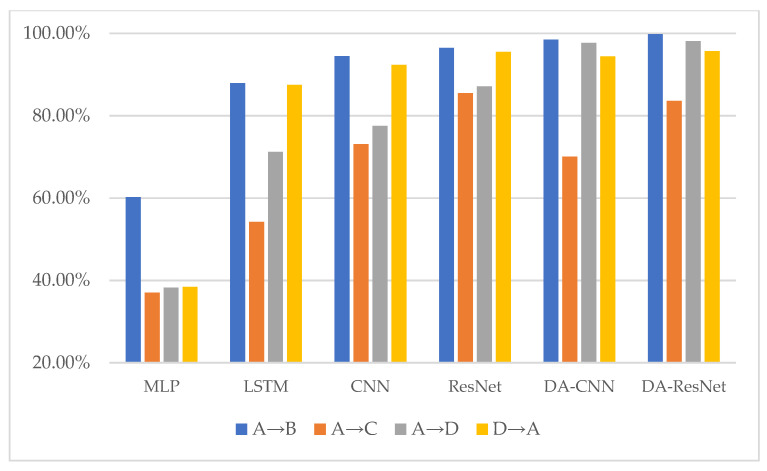
The F1-scores of the six methods on various transfer diagnostic tasks.

The diagnostic results of six methods are given in Table 5, and their corresponding histogram is shown in Figure 6. The F1-scores of MLP, BiLSTM, CNN, ResNet, DA-CNN, and DA-ResNet are shown in Table 6. The histogram of the F1-scores is shown in Figure 7. In Table 5, the capital letters A, B, C, and D are denoted by the fault degrees (also named datasets under the same defect size). For the detailed sizes, are refer to Table 2. For example, the fault degree A is the source domain and B is the target domain.

The F1-score is a tool that evaluates the accuracy of predictions and takes into account whether intelligent diagnostic methods have a preference for diagnostic performance in different categories. The formulas of the F1-score are given as follows:

(5) $$Precision=\frac{TP}{TP+FP}$$

(6) $$Recall=\frac{TP}{TP+FN}$$

(7) $$F1=\frac{2\times Precision\times Recall}{Precision+Recall}$$

in which *TP* is denoted as the predicted positive class, *FP* is the predicted positive class of error, and *FN* represents the predicted negative class of error. The parameter definitions of the above formulas are shown as follows: precision and recall represent accuracy and recall, respectively, and *F*1 is the F1-score. The F1-score is introduced to evaluate the diagnostic property of MLP, BiLSTM, CNN, ResNet, DA-CNN, and DA-ResNet in diagnostic tasks.

The key to this case is to use one defect size of the faulty element of bearings to diagnose the other fault types. The diagnosis results of the MLP, BiLSTM, CNN, ResNet, and DA-CNN are given in Table 5. In Table 5, the capital letters A, B, C, and D denote the fault degrees (also named datasets under the same defect size). The detailed sizes can be referred to in Table 2. For example, the fault degree A is a training sample, and B is used to test the sample. The average accuracy of the compared methods is 48.9%, 74.27%, 85.43%, 88.73%, and 91.83%. However, the proposed method result is 94.22%, which is superior to the comparison methods in four diagnostic tasks. Especially in the task from A to C, the lowest accuracy is half of the highest diagnostic value. The result can predict the MLP, and BiLSTM methods cannot separate the unknown label samples. CNN and ResNet can separate parts of unknown label samples, Nevertheless, without solving the problem of domain adaptation that model would not lead to a good result. The structure characteristic of ResNet is residual connections; a residual block is introduced to solve the problem of the accuracy decrease as a result of the increase of network depth. Furthermore, the residual block can keep the performance of models while the depth of the model increases. On the contrary, the characteristic of conventional networks is that accuracy will maintain a certain value and degrade rapidly as the network depth increases; meanwhile, more layers are added will bring more high training error. From Table 6, the highest F1-score values are used in bold font in the table. The performance of MLP in four diagnostic tasks is not good, and the proposed method has the highest F1-score in three diagnostic tasks. Combined with the accuracy of the DA-ResNet, it can be verified that the diagnostic property is superior to the other diagnostic methods. 

**Figure 8:**
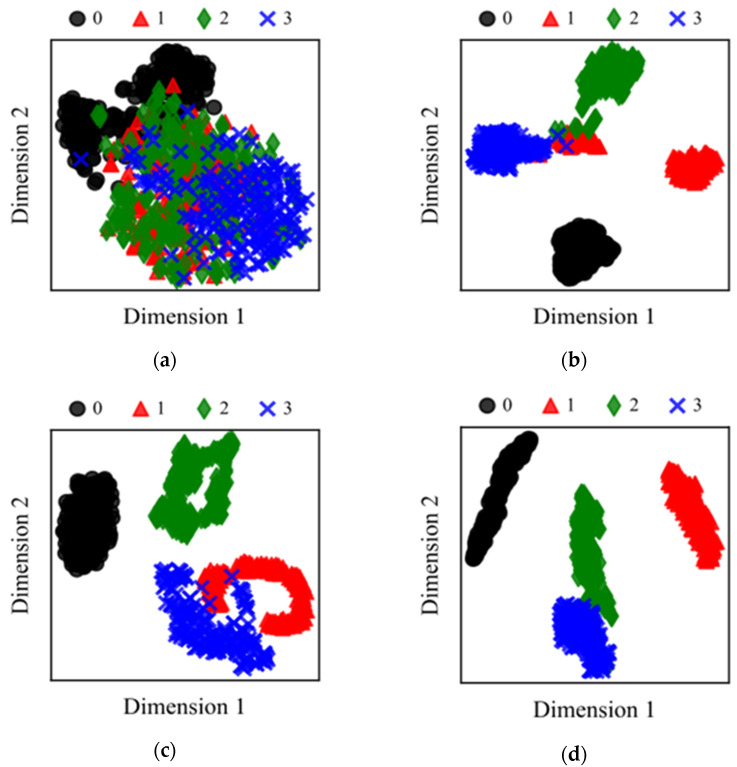

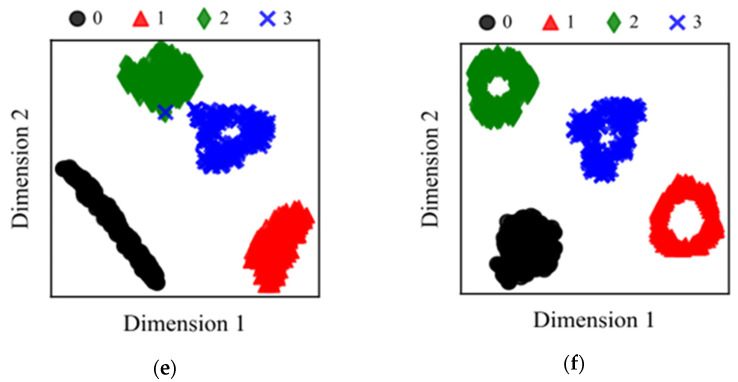
Feature representation of the methods in task A→B. (**a**) MLP; (**b**) BiLSTM; (**c**) CNN; (**d**) ResNet; (**e**) DA-CNN; (**f**) DA-ResNet.

**Figure 9:**
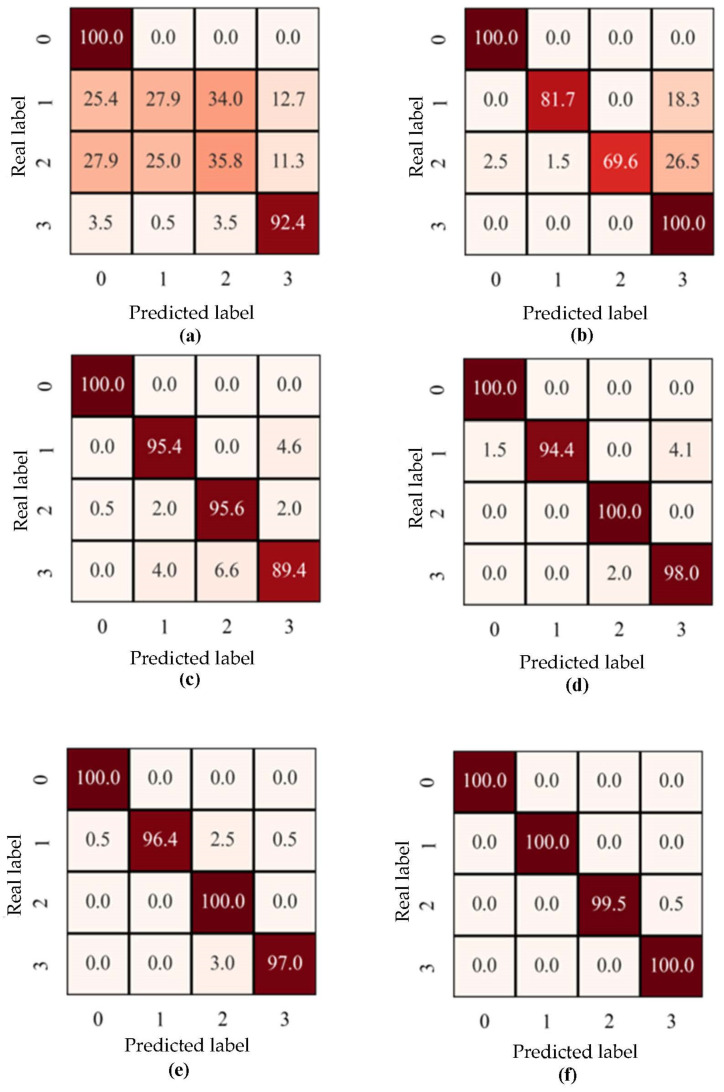
Confusion matrix demonstrating classification performance of methods in the task A→B. (**a**) MLP; (**b**) BiLSTM; (**c**) CNN; (**d**) ResNet; (**e**) DA-CNN; (**f**) DA-ResNet.

To compare the results of six methods, t-Distributed Stochastic Neighbor Embedding (t-SNE) [43] is introduced to visualize the operating results. Four colors represent four healthy conditions of roller bearings. As shown in Figure 8, the four colors are completely mixed together, which is obtained by MLP. Comparing CNN and ResNet, the blue and red colors are mixed, which is obtained by using the CNN model, and the result is worse than ResNet’s result. The confusion matrix of diagnostic results of MLP, BiLSTM, CNN, ResNet, DA-CNN, and DA-ResNet is shown in Figure 9. It is obvious that the multi-layer method MLP has not solved the domain adaption problem of source and target domains, only in the shared part with the recognition accuracy being close to 95%. The non-shared part from the target domain is mixed with the other classes and the average accuracy is 15%. The confusion matrix of BiLSTM is better than MLP; however, the second class is used to predict the same class with a low diagnostic accuracy of 69.6%. Comparing CNN with DA-CNN methods means that the MMD principle can decrease the discrepancy of the source and target domains. The same result is obtained from ResNet and DA-ResNet. Then, the diagnostic accuracy of DA-CNN is lower than DA-ResNet. The result indicates that the conventional network accuracy will maintain a certain value and degrade rapidly as the network depth increases. Meanwhile, more layers are added which will bring a higher training error. 

### 4.4. Transfer Diagnosis of Multi-Source Signal

To testify the multi-source data necessity in fault diagnosis, the fault degrees A and D are applied to check the performance of the proposed model. We prepared the single channel vibration signal of roller bearings, the acoustic signal, and the vibro-acoustic multi-source data to train the six models. The necessity of multi-source data is verified by the diagnostic accuracy of the six models in the fault diagnosis. 

**Figure 10:**
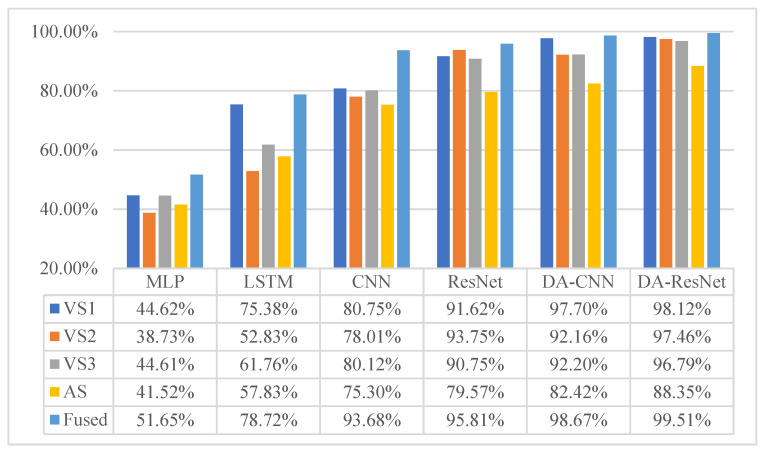
The accuracy of the six methods on various signals.

The diagnosis accuracy of six models on various signals and the multi-source signal is exhibited in Table 6. A single vibration or acoustic signal is used to train the model and then diagnosis fault degree D. The diagnosis accuracy of the six models on various signals and the multi-source signal is shown in Table 6 and is visualized in Figure 10. The accuracy of the vertical direction of vibration signals is from 44.62% to 98.12%. The difference value is approximately 53.5%, which is bigger than the accuracy of the MLP model. For the horizontal direction vibration signals, the difference value is 58.73%, which is bigger than the vertical direction accuracy. In the acoustic signal case, the difference between the highest and lowest is 46.83%, which indicates that acoustic signals include more fault information than vibration signals. The proposed model achieves the highest recognition accuracy in a single channel or multi-source signals. Furthermore, we can calculate the difference values between the highest and lowest accuracy values from six channels in Figure 10. The values range from 11.16% to 25.89%, and it can be concluded that the multi-source data could improve the diagnosis accuracy to a certain extent.

## 5. Discussion

This paper has achieved fault diagnosis under different failure degrees in one machine. From the accuracy results of the cross-domain fault diagnosis and transfer diagnosis of multi-source signals, the proposed model can obtain the highest recognition accuracy. However, in some transfer tasks, the accuracy value cannot achieve a higher accuracy. For example, in the task from A to C, the value only reaches 83.5% by DA-ResNet, which may guess the extracted features of the failure degree C are a little similar to failure degree A, and then the distribution between failure degree C and failure degree A has not decreased in minimum value. Therefore, the proposed model should be changed to balance the recognition accuracy in four transfer tasks. 

## 6. Conclusions

In this paper, we look at the problem of the distribution of datasets being different in obtaining bearings’ fault data, a fault diagnosis method based on MMD named DA-ResNet is proposed. At the same time, the vibration and acoustic data are sampled synchronously as the input term in the proposed model. The multi-source data can perfect the mechanical equipment information of rotating machinery and the proposed method can improve the generalization ability of the model. From the first experimental case, the comparison results of MLP, BiLSTM, CNN, ResNet, DA-CNN, and DA-ResNet are given through the confusion matrix. The highest diagnosis accuracy is obtained by DA-ResNet. In the last case, the necessity of multi-source data is verified by the histogram, and the tool could improve the diagnosis accuracy to a certain extent. Finally, the performance of the proposed method through related experiments could further verify the effectiveness and feasibility of this paper.

The proposed method achieves fault diagnosis from cross-domain by using vibration and acoustic data. In future work, the more physical quantities are considered as the input of the model, maybe the higher accuracy is acquired. The importance of this issue is to study the relation in physical quantities for further research to get high accuracy of faulty recognition. The research object is the roller bearing, which is a simple structure to extract features in fault diagnosis. Therefore, the next step of this paper is to change complex machinery parts to verify the performance of the proposed model. The proposed model may be introduced to engineering applications rather than in experimental test rigs if the complex machinery parts case will succeed.

## Figures and Tables

**Table 1:** Parameters of roller bearings.

Bearing Type	Ball Number *Z*	Pitch Diameter *D*	Ball Diameter *d*	Contact Angle *θ*	Inner Race Diameter	Outer Race Diameter
NU205E	13	38.9 mm	7.5 mm	0^o^	25 mm	52 mm

**Table 2:** Defect sizes of inner race, outer race, and roller.

Dataset	Fault Types	Defect Width (mm)	Labels	Dataset	Fault Types	Defect Width (mm)	Labels
A	N	0	0	C	N	0	0
	IR-I	0.43	1		IR-III	1.56	7
	OR-I	0.42	2		OR-III	1.55	8
	RO-I	0.49	3		RO-III	1.73	9
B	N	0	0	D	N	0	0
	IR-II	1.01	4		IR-IV	2.03	10
	OR-II	0.86	5		OR-IV	1.97	11
	RO-II	1.16	6		OR-IV	2.12	12

**Table 3:** Detailed parameters of the DA-ResNet model.

Layer (Type)	Output Shape	Param #
inputs1 (Input layer)	(4096, 1)	0
c0 (Conv1D)	(1024, 4)	20
c11 (Conv1D)	(512, 8)	104
c12 (Conv1D)	(512, 8)	200
add_1_2 (Add)	(512, 8)	0
x1p (Max-pooling1D)	(256, 8)	0
c21 (Conv1D)	(256, 16)	400
c22 (Conv1D)	(256, 16)	784
add_2_2 (Add)	(256, 16)	0
x2p (Max-pooling1D)	(128, 16)	0
c31 (Conv1D)	(128, 32)	1568
c32 (Conv1D)	(128, 32)	3104
add_3_2 (Add)	(128, 32)	0
x3p (Max-pooling1D)	(64, 32)	0
flatten (Flatten)	(2048)	0
out (Dense)	(128)	262,272

**Table 4:** Detailed information of four models.

Model	Embedding	Characteristics	Params
MLP	None	Fully connection	528,554
BiLSTM	Convolution	LSTM	16,704
CNN	None	Convolution	65,820
ResNet	None	Residual block	268,452

**Table 5:** The accuracy of six methods for four transfer diagnostic tasks.

Model	A→B	A→C	A→D	D→A
MLP	64.00%	41.87%	44.62%	45.12%
BiLSTM	87.83%	59.25%	75.38%	74.62%
CNN	95.10%	73.35%	80.75%	92.50%
ResNet	96.50%	71.3%	91.62%	95.50%
DA-CNN	98.37%	76.75%	97.70%	94.50%
DA-ResNet	**99.87**%	**83.5**%	**98.12**%	**95.40**%

**Table 6:** The F1-score of six methods for four transfer diagnostic tasks.

Model	A→B	A→C	A→D	D→A
MLP	60.25%	37.03%	38.22%	38.45%
BiLSTM	87.88%	54.22%	71.26%	87.49%
CNN	94.50%	73.10%	77.53%	92.34%
ResNet	96.50%	85.50%	87.10%	95.50%
DA-CNN	98.46%	70.07%	97.70%	94.40%
DA-ResNet	**99.80**%	**83.62**%	**98.10**%	**95.70**%

## Data Availability

The data presented in this study are available on request from the corresponding author.
